# Quantitative trait loci influencing forking defects in an outbred pedigree of loblolly pine

**DOI:** 10.1186/s12863-016-0446-6

**Published:** 2016-10-18

**Authors:** Jin S. Xiong, Steven E. McKeand, Fikret Isik, Jill Wegrzyn, David B. Neale, Zhao-Bang Zeng, Luciano da Costa e Silva, Ross W. Whetten

**Affiliations:** 1 Cooperative Tree Improvement Program, Department of Forestry & Environmental Resources, North Carolina State University, Raleigh, USA; 2 Present address: Dow AgroSciences, Indianapolis, IN USA; 3 Department of Plant Sciences, University of California, Davis, USA; 4 Present address: University of Connecticut, Storrs, CT USA; 5 Department of Statistics and Bioinformatics Research Center, North Carolina State University, Raleigh, NC USA; 6 Universidade de São Paulo – ESALQ, Saõ Paulo, Brazil; 7 Present address: SAS Institute, Cary, NC USA; 8 Department of Forestry & Environmental Resources, North Carolina State University, Raleigh, NC 27695-8008 USA

**Keywords:** QTL, Stem forking, Outbred, *Pinus taeda*, MAS, Tree breeding, Wood quality

## Abstract

**Background:**

The use of wood as an industrial raw material has led to development of plantation forestry, in which trees are planted, managed, and harvested as crops. The productivity of such plantations often exceeds that of less-intensively-managed forests, and land managers have the option of choosing specific planting stock to produce specific types of wood for industrial use. Stem forking, or division of the stem into two or more stems of roughly equal size, is a character trait important in determining the quality of the stem for production of solid wood products. This trait typically has very low individual-tree heritability, but can be more accurately assessed in clonally-replicated plantings where each genotype is represented by several individual trees. We report results from a quantitative trait mapping experiment in a clonally-replicated full-sibling family of loblolly pine (*Pinus taeda* L.).

**Results:**

Quantitative trait loci influencing forking defects were identified in an outbred full-sibling family of loblolly pine, using single-nucleotide polymorphism markers. Genetic markers in this family segregated either in 1:2:1 (F2 intercross-like segregation) or 1:1 ratio (backcross-like segregation). An integrated linkage map combining markers with different segregation ratios was assembled for this full-sib family, and a total of 409 SNP markers were mapped on 12 linkage groups, covering 1622 cM. Two and three trait loci were identified for forking and ramicorn branch traits, respectively, using the interval mapping method. Three trait loci were detected for both traits using multiple-trait analysis.

**Conclusions:**

The detection of three loci for forking and ramicorn branching in a multiple-trait analysis could mean that there are genes with pleiotropic effects on both traits, or that separate genes affecting different traits are clustered together. The detection of genetic loci associated with variation in stem quality traits in this study supports the hypothesis that marker-assisted selection can be used to decrease the rate of stem defects in breeding populations of loblolly pine.

**Electronic supplementary material:**

The online version of this article (doi:10.1186/s12863-016-0446-6) contains supplementary material, which is available to authorized users.

## Background

During the past 20 years, modern molecular marker technologies and genomic tools have been developed to accelerate the tree breeding process. The Conifer Translational Genomics Network Coordinated Agricultural Project (CTGN CAP) [1] had the objective of bringing genomics-based breeding to application in major forest tree improvement programs in the US. The CTGN CAP applied Illumina single-nucleotide polymorphism (SNP) genotyping technology to develop SNP makers for loblolly pine (*Pinus taeda* L.), slash pine (*Pinus elliottii* Engelm.), and Douglas-fir, (*Pseudotsuga menziesii* (Mirb.) Franco) [1]. The availability of SNP markers and efficient genotyping technology allows constructing dense linkage maps and accelerates studies on inheritance and genetic architecture of complex traits at the individual quantitative trait locus level. It also created the opportunities for large-scale genomic research in these commercially important forest tree species.

Loblolly pine is an important forest tree species in the southern United States. Tree improvement programs in loblolly pine have greatly increased the wood productivity over the last 50 years [2]. The improvement in tree growth rate results in much greater yields but also increases wood quality issues, which have drawn the attention of tree breeders in recent years [3–5]. Stem forking and ramicorn branching are examples of wood quality problems that are unfavorably correlated with height growth in loblolly pine [3]. A stem fork is a separation of the trunk into two or more stems of similar sizes, and a ramicorn branch is a branch with at least twice the diameter and a substantially steeper angle than other branches in the same whorl. Stem forking and ramicorn branching can be grouped together as stem forking defects, and these defects decrease the wood quality and the commercial value of timber in loblolly pine plantations.

A better understanding of the genetic architecture of forking defects is required in order to develop trees with lower frequency of forking defects through a selective breeding program. Our previous studies have shown that forking defect is under moderate genetic control at the family level, and shows strong genetic variation within full-sibling (full-sib) families [3, 6]. Repeatability of full-sib family mean forking value was 0.59 across based on 8000 full-sib progeny from an extensive set of a partial-diallel mating designs [3] and the clone-mean repeatability for stem forking was 0.67 in a clonally-replicated trial [6]. However, the individual-tree heritability was very low (0.06) in the population-wide survey [3]. Estimates of heritability for binary traits can be biased due to violations of the underlying assumptions [7], but this estimate shows the difficulty breeders face in trying to select individuals with a reduced frequency of stem quality defects. It would be beneficial to have marker-assisted technology for use in the improvement of forking defects, due to the low individual heritability and the difficulty in assessment of such traits at early ages. Detection of association between molecular markers and forking defects will help to understand the genetic architecture of these traits and will allow further application of markers in those breeding programs that target the improvement of forking defects.

Quantitative Trait Loci (QTL) related to wood properties, growth, adaptation, and drought-tolerance have been reported in pines [8]. Significant QTL were identified for wood specific gravity in an outbred pedigree of loblolly pine [9]. Sewell et al. [10] reported QTL related to the physical and chemical properties of wood with a small population of loblolly pine. Remington and O’Malley [11] studied embryonic viability loci in offspring from self-pollination of an individual loblolly pine, and identified 19 loci showing moderately deleterious to lethal embryonic effects. Neale et al. [12] summarized results of experiments that associated QTL with wood properties of loblolly pine. Brown et al. [13] verified that there are QTL influencing wood property traits in loblolly pine among multiple populations. However, to the best of our knowledge, there are no published studies of the genetic architecture of stem forking defects in loblolly pine.

We report analysis of the genetic architecture of forking traits in a clonally-replicated loblolly pine full-sibling family with 217 progeny genotypes each replicated up to 10 times at each of three locations. The specific objectives of the current study were: i) to construct a genetic linkage map for this loblolly pine family using SNP markers; ii) to identify QTLs associated with stem forking traits; and iii) to develop a marker-assisted breeding strategy to improve forking traits in loblolly pine.

## Methods

### Plant material and phenotyping

Clonally-replicated loblolly pine progeny from an outbred full-sibling family provided by MeadWestvaco (now ArborGen) were used in this study. Height, diameter at breast height (DBH), ramicorn branching, stem forking (fork), branch angle, and stem straightness (Strt) were measured at age six and age seven years as previously described [6]. Forking and ramicorn branching were scored as binary traits; each individual tree that showed a defect was scored as 1, while defect-free individuals were scored as 0. The volume of each individual tree was calculated according to the equation developed by Goebel and Warner [14]. Data were collected for 217 progeny (clones) of this full-sibling family, with up to 10 replications (ramets) of every clone at each location.

### Phenotypic data analysis

Stem forking and ramicorn branching are threshold characters with only two phenotypic classes, and are assumed to have an underlying continuous variation of liability [15]. The generalized linear mixed model with a logit link function was used to analyze the forking traits [6]:

$$ {\eta}_{ijk}= \log \left(\frac{\pi }{1-\pi}\right)=\mu +{t}_i+b{(t)}_{j(i)}+{c}_k+t{c}_{ik}+{e}_{ijk} $$

where

*η*_*ijk*_ is the link function;

π is the proportion of stem forking or ramicorn branches among ramets of a clone;

μ is the conditional mean;

t_i_ is the *i*th fixed test environment (location) effect;

b(t)_j(i)_ is the fixed effect of the *j*th block within *i*th test;

c_k_ is the random clone effect of the *k*th clone;

tc_ik_ is the random interaction effect of the *i*th test by the *k*th clone;

e _ijk_ is the random error term associated with the *k*th clone observation in the j*th* block of the i*th* test site.

Height, DBH, volume, straightness and branch angle were analyzed as quantitative traits. A general linear mixed model was used to analyze those traits. The model has been published in a previous paper [6].

Estimated genetic values of the clones from the genetic models were used as phenotypes for the QTL study.

### SNP genotyping

Needle tissue was collected from one ramet (genetically identical copy of a tree) of each of 217 full-sib progeny genotypes in the field when they were six and seven years old, and from ramets of the parent genotypes growing in the MeadWestvaco seed orchard. Total genomic DNA was isolated from the needles using a DNeasy-96 Plant Kit (Qiagen) following the manufacturer’s protocol. SNPs derived from the Allele Discovery of Economic Pine Traits project (ADEPT2; http://dendrome.ucdavis.edu/adept2/) were used. Further information regarding SNP discovery and annotation is available elsewhere [16]. Genotyping of SNPs utilizing the Infinium platform was carried out at the University of California Davis Genome Center. Arrays were imaged on a Bead Array reader (Illumina, San Diego CA, USA) and genotype calling was performed using BeadStudio v. 3.1.3.0 (Illumina), as previous described [16].

### Genetic map

The input marker data file used in the analysis was coded according to the segregation patterns described in Wu et al. [17]. Non-segregating loci were removed, and the marker data set with all three types of marker segregation patterns (B3.7 for F2 intercross like-markers, D1.10 for backcross like-markers from parent 1, and D2.15 for backcross like-marker from parent 2), as shown in Table 1 were used to construct an integrated map using the OneMap software [18]. Inclusion of all three types of markers (B3.7, D1.10 and D2.15) into the same linkage group (denoted here as an integrated map) was possible because the software uses multi-point maximum likelihood estimates of recombination fraction [18].

**Table 1:** Summary of scored markers

Cross type	Parent cross	Offspring genotypes observed	Segregation	# of markers	Configurations
1	aa × aa	a	-	3075	non-informative
2	aa × bb	ab	-	148	non-informative
B3.7	ab × ab	a, ab, b	1:2:1	304	F2 intercross
D1.10	ab × aa	a, ab	1:1	552	Backcross 1
D2.15	aa × ab	a, ab	1:1	696	Backcross 2

Three types of markers (B3.7, D1.10, and D2.15) were used to construct a linkage map

Pair-wise recombination fractions between markers were estimated via the two-point maximum likelihood estimation method, and markers were then assigned to linkage groups provided that adjacent markers had a minimum logarithm of odds (LOD) score of six for the linkage test and maximum recombination fraction (MRF) of 0.45. Marker order within each linkage group was first determined for the five most informative markers (B3.7 markers), and subsequently other markers were added sequentially, provided that they had a minimum LOD of 3 for the linkage test.

Each linkage group had the marker order checked for possible false positive linkages using the recombination fraction heat map produced by the rf.graph.table() function of the OneMap package [18], and adjustments to the marker order were carried out according to the plotting diagnosis. SNPs that were mapped to the same position in the initial linkage groups were filtered out prior to the estimation of the final linkage groups to remove redundant information. The genetic distance function of Haldane [19] was used to calculate map distance. Linkage group numbers and orientation of the map were defined based on the consensus genetic map of *Pinus taeda* [20]. The graph of the linkage map was built using the software MapChart [21].

### QTL analysis

QTL analysis was carried out utilizing the PROC QTL procedure [22] of SAS software [23]. Genetic values of the clones estimated from the genetic models (as described in the Phenotypic Data Analysis section) were used as pseudo-phenotypes. The linkage map was used to map putative QTLs. The entire genome was scanned on one centiMorgan (cM) window using the interval mapping [24] method under the following QTL model [25]:

$$ {y}_j={Z}_j\beta +{\varepsilon}_j $$

Where

y_j_ is the phenotype of the j*th* individual (j = 1,…,n),

*β* is a vector for QTL effects of a putative locus,

Z_j_ is a vector for the genotype indicator variable.

*ε*_*j*_ is residual, N(0, σ^2^).

The method of maximum likelihood were used for interval mapping QTL of single trait [24, 26, 27], based on a four-way cross model of genetic segregation. A Bayesian method was used for multi-trait joint mapping [22].

The likelihood ratio test (LRT) statistic was converted to a log of odds LOD score by the formula $\mathrm{L}\mathrm{O}\mathrm{D}=\frac{1}{2}\left({ \log}_{10}\mathrm{e}\right)\ \mathrm{L}\mathrm{R}\mathrm{T}=0.217\ \mathrm{L}\mathrm{R}\mathrm{T}$ Permutation tests were used to find the genome-wide threshold value for the LOD score [28]. The measurements of phenotype and genotype were shuffled among individuals to generate a permuted sample of the data (to simulate the null hypothesis of no association between genotype and phenotype). Each permuted dataset was used to perform interval mapping analysis, and the maximum LOD score over all analysis points for each of the permuted dataset was recorded. These LOD score values were then ordered, and their 95 percentile is the empirical estimated genome-wide threshold value. When the LOD score estimated from the original data in a genomic region is higher than the genome-wide threshold value, a QTL is declared. One thousand permutations were used in the estimation of the genome-wide threshold.

## Results

### Data summary

The proportion of forking ranged from 13 % to 21 % across four test sites with a mean of 17 %. Ramicorn branching incidence averaged 24 % and varied from 18 % to 31 % over all sites. Significant differences were found among clones for forking and ramicorn branching. The forking proportion among clones ranged from 0 % to 73 %, and ramicorn percentages varied from 3 % to 50 % among clones. Frequency distributions of forking and ramicorn branching have been published in our previous paper [6]. The clone-mean repeatability for forking was 0.86, and the clone-mean repeatability for ramicorn branching is 0.67.

Of the 4775 scored SNPs, 1552 SNPs were informative (i.e. were polymorphic among the offspring): 552 of type D1.10 (backcross 1), 696 of D2.10 (backcross 2), and 304 of B3.7 (F2 intercross) (Table 1). There were 3,223 SNP loci that were homozygous in both parents, so those markers were not informative for the analysis. Chi-square (*χ* ^2^) tests were applied to verify the observed and expected Mendelian segregation ratios of 1:2:1 (F2) and 1:1 (backcross 1 and 2). Within the set of informative markers, 439 markers showed segregation distortion (*P* < 0.05): 67 in the F2 set, 120 in the backcross 1, and 252 in the backcross 2. These markers were omitted from further analysis. A total of 1113 SNPs were used in linkage mapping analysis.

### Linkage map

**Fig. 1:**
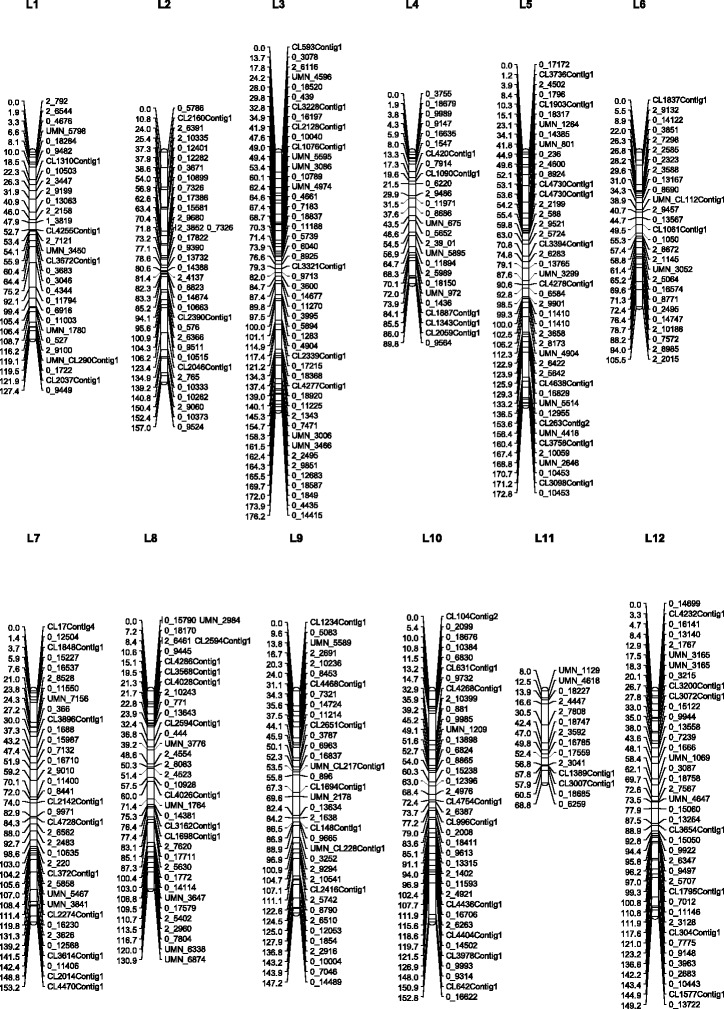
Final genetic linkage map with 12 groups

When using a minimum LOD value of 6.0 and a maximum recombination fraction of 0.45 in grouping the markers, 12 linkage groups were obtained. The redundant SNPs (markers that map in exactly the same position) were removed prior to further analysis; this filtering step removed 704 of the 1113 markers. The remaining 409 markers were used to define an initial linkage map with total length of 1622 cM in 12 groups (Fig. 1). The linkage groups were aligned with the consensus genetic linkage map of *Pinus taeda* [20] using markers found in both maps. Linkage group 3 is the largest group with 48 markers, and group 11 is the smallest one with only 14 markers. The average distance between adjacent markers over all groups is 3.9 cM.

### Detection of QTL

The QTL mapping analysis used the linkage map (Fig. 1) as the basis for detecting genomic regions controlling traits.

**Fig. 2:**
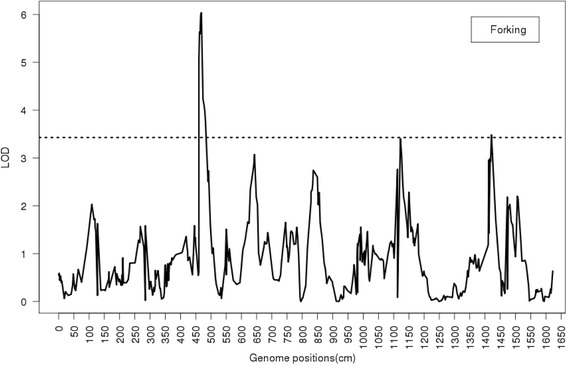
Profiles of the logarithm of odds (LOD) ratio test statistic from maximum likelihood (ML) methods for forking. The 12 groups are merged into a single line on the horizontal axis. The dashed line through the middle of the graph represents the LOD threshold value of 3.43 determined by permutation analysis

The permutation tests yielded a LOD score threshold for the stem forking trait of 3.43 at a statistical significance level of α = 0.05, so 3.43 was used as the threshold to detect QTL for stem forking with genome-wide significance. Across the whole genome, there were two peaks over the threshold: one at the position of 468.51 cM and the other at 1421.11 cM (Fig. 2). The closest markers to these two QTL were 0_1547 and 2_4447, with LOD scores of 6.038 and 3.483, respectively (Table 2). These two QTL account for 84–86 % of the total variation of clonal estimated genetic values (EGV) for forking, according to the variance component analysis.

**Table 2:** Interval mapping parameter estimates for QTL controlling forking

QTL	Nearest marker	Group	LOD	Position (cM)	μ	a__1_	a__2_	σ^2^
FQ1	0_1547	4	6.038	468.51	0.154	0.026	0.020	0.007
FQ2	2_4447	11	3.483	1421.11	0.156	−0.019	−0.017	0.007

Parameter μ is the mean, a__1_ is the additive effect from parent 1, a__2_ is the additive effect from parent 2, and σ^2^ is the residual variance. (Standard errors of a_1 and a_1 for both QTL were 0.006.)

**Fig. 3:**
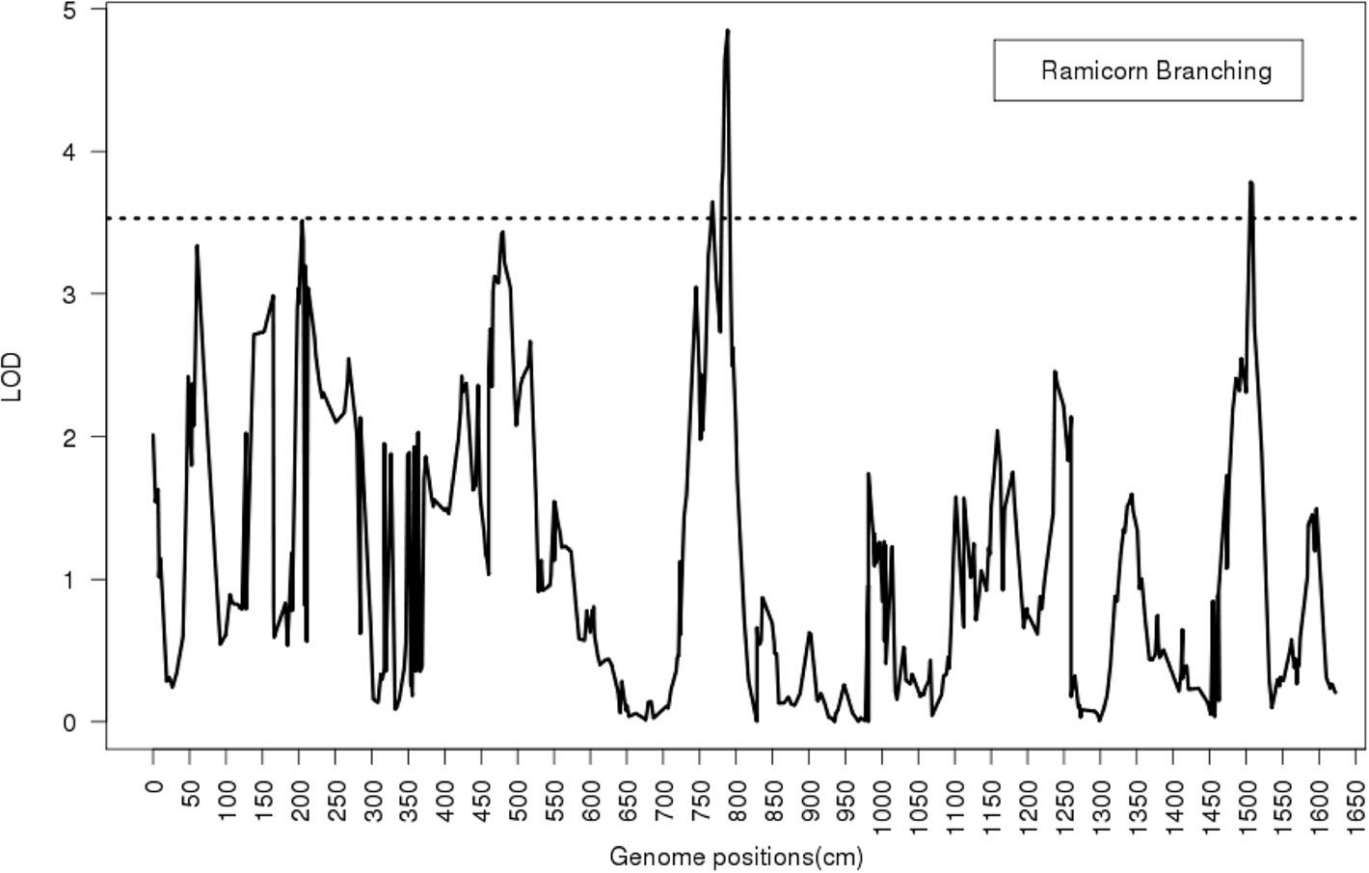
Profiles of the logarithm of odds (LOD) ratio test statistic from ML for ramicorn branching. The 12 groups are merged into a single line on the horizontal axis. The dashed line through the middle of the graph represents the LOD threshold value of 3.53

For ramicorn branching, three QTL exceed the LOD score threshold (3.53) at positions 767.74, 788.28, and 1508.38 cM (Fig. 3). The SNPs near these QTL were 0_13567 & 2_5064 from group 6, and 0_9944 from group 12 (Table 3). These QTL explained 82–87 % of the total EGV of ramicorn branching.

**Table 3:** Interval mapping parameter estimates for QTL controlling ramicorn branching

QTL	Nearest Marker	Group	LOD	Position (cM)	μ	a__1_	a__2_	σ^2^
RQ1	0_9944	12	3.773	1508.38	0.442	−0.004	−0.020	0.005
RQ2	0_13567	6	3.645	767.74	0.441	−0.015	0.016	0.005
RQ3	2_5064	6	4.848	788.28	0.443	−0.016	0.017	0.005

Parameter μ is the mean, a__1_ is the additive effect from parent 1, a__2_ is the additive effect from parent 2, and σ^2^ is the residual variance. (Standard errors of a_1 and a_1 for three QTL were 0.005.)

QTL were also found for growth traits. Five QTL were identified for volume, on linkage group 1, 2, 4, 9 and 10 (Table 4), when using a LOD threshold of 3.61. Of the total variation of volume, QTL effects explained 2.7–5.7 %. Three QTL were found for height, at positions 164.68, 990.06 and 1412.55 cM (Table 4). Additionally, one QTL was found for straightness, and the nearest marker to it was 0_9944 (Table 4). No QTL was found for branch angle.

**Table 4:** QTLs identified associated with stem volume, tree height, and stem straightness

Trait	LOD Threshold	# of QTL	Linkage group	Loci	SNP markers	H^2^ (%)
Volume	3.61	6	9, 2, 10, 1, 1, 4	1195.02, 212.62, 1412.55, 54.09, 1.91, 480.13	0_13634, 0_10663, 0_16622, UMN_3450, 2_6544, CL1090Contig1	2.7–5.7
Height	3.82	3	2, 10, 8	164.68, 1412.55, 990.06	0_12401, 0_16622, 2_6461	0.84–6.42
Straightness	3.51	1	12	1508.38	0_9944	3.3–9

H^2^ is the heritability of the QTL for given trait; it is the ratio of additive variance of QTL to total variance

**Fig. 4:**
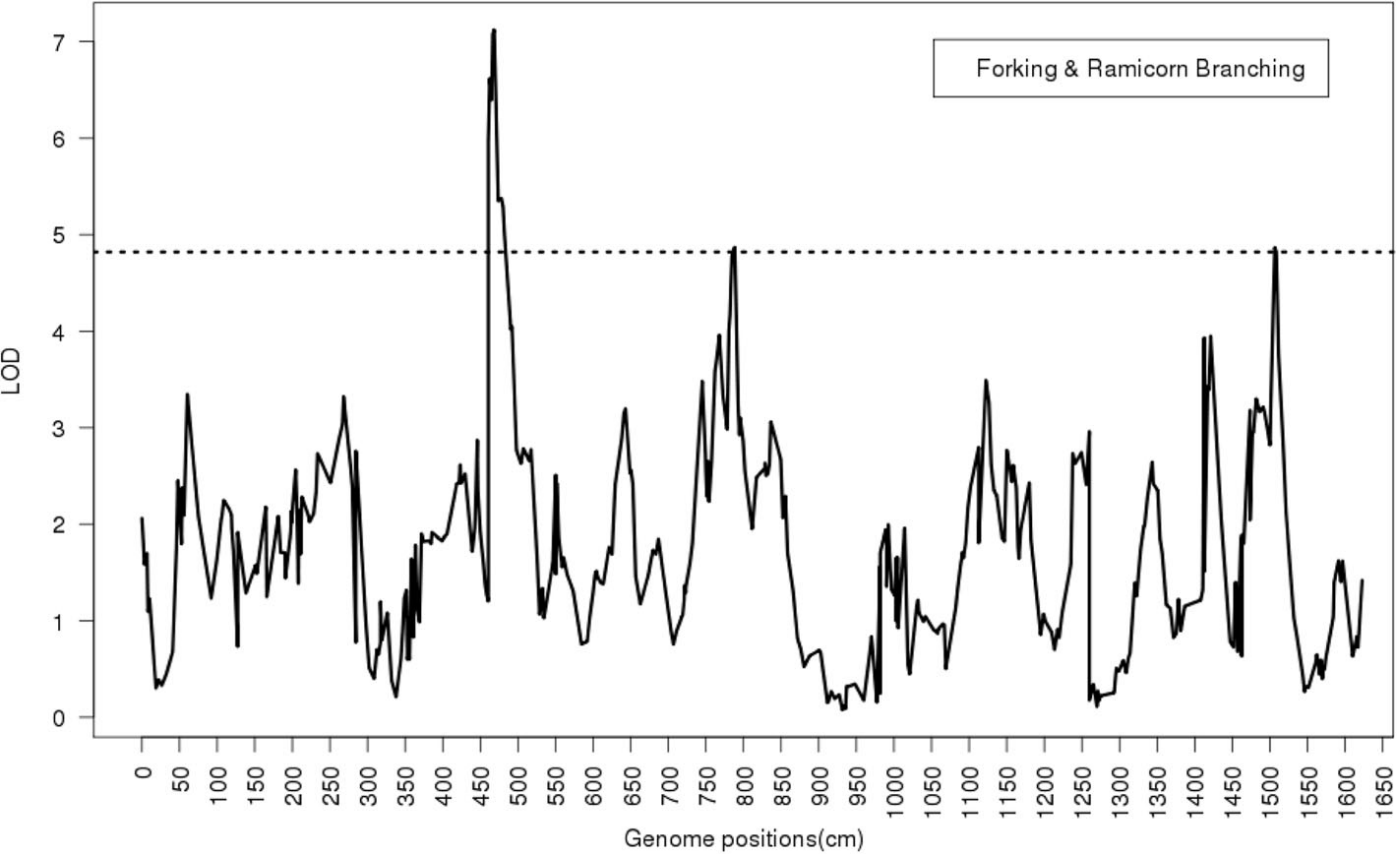
Profile of the logarithm of odds (LOD) ratio test statistic for the joint analysis of forking and ramicorn branching. The 12 groups are merged into a single line on the horizontal axis. The dashed horizontal line represents the LOD threshold value of 4.82

Through the multiple trait analysis, QTL controlling pairs of traits were identified. From the joint analysis on forking and ramicorn branching, three QTL were detected at position 468.51, 788.28 and 1508.38 cM (Fig. 4). Markers 0_9944, 2_5064 and 0_1547 are the closest SNPs, and could be used as surrogate markers to identify the QTL controlling both traits.

## Discussion

### Linkage map

The linkage map in this study was aligned to the consensus genetic map for *Pinus taeda* [20]. The consensus map was constructed using 1251 individuals from four different populations [20], and currently serves as the reference for placement of SNP markers in the *Pinus taeda* linkage map. The similarity of marker orders and distance are high between the two maps. With 275 common markers from both maps, the average correlation of marker distance within linkage groups is 0.97. The linkage disequilibrium (LD) and population size determine the density of genetic markers needed to construct a linage map. In this study of a full-sib family population of 217 individuals, linkage disequilibrium (LD) is expected to be high. Consistent with that expectation, a large proportion of the SNP markers (704 of 1113) were found to be redundant with other markers in building the genetic linkage map. The 409 mapped SNP markers should have substantial predictive abilities for forking traits for the progeny from this single family.

Few studies have previously reported QTL mapping for either stem form or branch architecture in forest trees. Shepherd et al. [29] reported that no QTL were detected for ramicorn branching or double leaders (forking) in *Pinus elliottii var. elliottii × Pinus caribaea var. hondurensis* hybrids. In Shepherd’s study, markers showing a segregation ratio of 3:1 were excluded from the analysis, in order to use the pseudo-testcross strategy of mapping [30]. As a result, the power to detect effects of QTL could be low, as a backcross design has only half the power of the F2 intercross design to detect small effect QTL [31]. In our study, markers from all three cross types were used to construct an integrated genetic linkage map that better represents the genome of loblolly pine and is more informative for QTL detection. To the best of our knowledge, this is the first published QTL mapping analysis of forking defects in loblolly pine, and the first study using cloned full-sibling progeny to increase the accuracy of phenotype assessment of forking defects in pine.

The QTL analysis through interval mapping has identified two and three QTL for forking and ramicorn branching, respectively, with strong additive value of QTL effects. This result indicates that forking traits may be controlled by a few genes with large effects in this family, although QTL mapping in a single full-sib family of 217 individuals is likely to both underestimate the true number of QTL and overestimate the magnitude of the effects of detected QTL [32, 33]. Our previous studies showed that forking is under genetic control at the family level in a broad sample of the loblolly pine population, with moderate family-mean heritability of 0.59–0.76 [3]. Forking defects were reported to be under strong genetic control at the cloned progeny mean level, with moderate to high clone mean repeatability of 0.67 to 0.86 [6]. The high heritability of forking traits at clone level are consistent with the high values of QTL effects of forking defects estimated in this study. While additional studies with larger numbers of clonally-replicated progeny from a larger sample of parents will be necessary to obtain more accurate estimates of the true numbers of loci that affect variation in stem quality traits and the magnitude of effects of individual loci, this study provides evidence that genetic control of loblolly pine stem defects does exist and supports the need for additional research in this area of wood quality.

Heritability plays an important role in determining the value of additive effects from significant QTL [30]. The majority of QTL mapping studies in loblolly pine reported in the literature used seedling trials with a single measurement per genotype, which have limited power to estimate genetic values of low-heritability traits [34]. Use of clonally replicated progeny for QTL studies allows more accurate assessment of traits with relatively low individual tree heritability. The repeatability will increase by using clonal materials from the same genotypes tested in different environments [35–37]. It was reported that a sample size of 200 would be needed to detect half of the additive genetic variance at α = 0.01, when the within-family heritability is 0.5 and five effective QTL control the trait [38]. With the sample size of 217 and clonal clone-mean repeatability of 0.67 to 0.86 for forking traits in this clonal trial, the power to estimate QTL locations and effects should be relatively strong in our study.

Growth traits such as height and diameter normally have low heritability in tree populations, and the detected QTL for height and volume show low estimated magnitudes of effects in this study. This observation is consistent with the hypothesis that complex quantitative traits in forest trees are typically controlled by many genes of relatively small effect [39]. Height and diameter growth in loblolly pine typically show relatively high genetic correlation [40], so it is not surprising that SNP 0_16622 was identified as associated with QTL affecting with both height and volume (calculated from height and diameter by the equation of Goebel and Warner [14]). SNP 0_9944 was identified as associated with Straightness as well as Ramicorn branching. Our previous study had shown that there is positive genetic correlation between ramicorn and straightness which means straighter trees had fewer ramicorn defects [6]. The favorable correlation suggested that straight trees have a tendency not to have forking defects, but an alternative explanation is that measurement crews tended to evaluate trees with stem forks or ramicorn branches trees as below average for straightness, which could cause a positive correlation between an absence of stem defects and straightness without an underlying common genetic mechanism.

The observation that joint analysis of ramicorn branching and stem forking detects three QTL associated with phenotypic variation in both traits is not surprising, because a moderately high genetic correlation was previously reported between these two traits (rg = 0.68) [3]. This observation is consistent with several hypotheses. Two alternate genetic hypotheses are (1) the same genes have pleiotropic effects on both stem forking and ramicorn branching, or (2) different genes affect the two traits but they are clustered together and cannot be resolved from each other in this size mapping population. An additional alternative is that stem forks and ramicorn branches are essentially a single biological phenomenon with phenotypic variation that varies continuously from one extreme (clearly a forked stem) to the other (clearly a ramicorn branch) across intermediate levels that might be scored differently by different observers. In any case, it is possible to decrease both forking defects simultaneously through selection, and the markers identified in this study will at least be useful for selection within pedigrees derived from the two parents of this mapping population. The QTL for forking and ramicorn branching could be used for marker assisted selection on both traits. However, the unfavorable correlations between forking defects and growth might cause negative effects on one trait when selecting for the other.

## Conclusions

Forking traits are usually difficult to measure consistently because individual foresters can evaluate the presence of a fork or ramicorn branch phenotype differently. Additionally, the individual-tree heritability of forking is very low, so conventional selection carried out based on observed phenotypes may not achieve desirable gains in decreasing forking defects. The detection of QTL and the associated molecular markers could be used for marker-assisted selection in tree improvement programs to decrease the incidence of stem forks and ramicorn branches. The QTL detected in this study are likely to account for at least half of the genetic variation of forking traits in this family after taking the known bias due to small population size into account [33], so selection of individuals within family on markers linked to these QTL will likely result in greater genetic gain than phenotypic selection alone. The two parents of this full-sib family are important elite parent lines in the NCSU Cooperative Tree Improvement Program, so significant markers identified from this full-sib family might have applications in Cooperative breeding populations.

In the third cycle of loblolly pine breeding, more focus has been put into within-family selection by using clonal tests [2]. Within-family selection can exploit the large amount of linkage disequilibrium created within full-sibling families, and clonal replication can capture non-additive genetic variation, which provides favorable conditions for marker-assisted selection (MAS) in loblolly pine [41]. Favorable loci segregating within families could be efficiently tagged by markers and used for marker-assisted within-family selection for elite individuals. Clonal testing could be an efficient way to track the inheritance and segregation of important QTL on an individual basis. Using the trait-associated markers to pre-screen individual clones would allow early selection of superior germplasm for either progeny-testing as potential parents or for clonal deployment in operational plantations [42].

The usage of MAS has been postulated to have a potential to accelerate introgression of desired genes into breeding populations and shorten the breeding cycles. This would be most relevant for traits controlled by individual genes with major effects, such as resistance to fusiform rust disease in loblolly pine, which has been shown to be controlled by individual genes with major effects on phenotype [43]. Moreover, the development of new technologies for high throughput genotyping makes the cost of MAS much lower than before, allowing more cost-effective applications of genotyping in tree breeding program. The proportion of variation in height and volume accounted for by QTL detected in this experiment is too small to justify marker-assisted selection for those traits. A genome-wide selection approach may be more useful for improving growth traits [44]. Several proof of concept studies on genome-wide selection in loblolly pine are encouraging [45–47].

The strategies of QTL mapping analysis in outbred populations described to date have used individual families within a single population to increase the degree of linkage disequilibrium, but results from single family analysis may have limited utility for MAS in other families. The recombination fraction will change in different genetic backgrounds, which will affect the confidence of QTL – marker association across families [38]. Linkage phase relationships between markers and QTL will differ among pedigrees, leading to questions about the generalization of QTL findings from experimental populations to breeding populations [44]. In addition, loblolly pine is a long-lived organism, so the consistency of QTL may vary across ages. Changes in QTL expression as trees age have already been reported in poplar for basal area [48] and Eucalyptus for multiple traits [49]. Plomion et al. [50] also found that different loci may be involved in the genetic control of height-growth at different ages in *Pinus pinaster*. Marker aided selection may also have limited application in cases of interaction between QTL and environment, unless a specific association of marker, trait, and environment is used to guide MAS. A QTL study in chemical contents found that there were significant differences among populations of loblolly pine from North Carolina and Oklahoma, and it was suggested that the QTL interacts with environmental location [51].

We assessed forking traits at four different sites in this study and did not find statistically significant interaction of sites and clones, so there was little evidence of genotype by environment interaction for forking [3]. In the presence of genotype by environment interaction effects, QTL analysis may need to be carried out in different environments to increase the reliability of MAS. The correlation of forking and ages was not assessed in this study, but a study in ash (*Fraxinus excelsior*) found that the proportion of forked trees was much higher in later ages than in the first four years growth [52]. If there is significant QTL by age interaction for forking traits, analysis of QTL at different ages may be useful, but need for this analysis should be based on evaluation of the economic impact of forking at later ages. Stem defects that occur high in the crown of loblolly pine trees have less economic impact on the value of the tree than do forks at positions closer to the ground.

In this study, putative QTL controlling forking defects were identified by analyzing a single family, so the immediate application of these results is limited to this family. The results of this study and our previous studies showed, however, that future studies are warranted because forking is clearly under genetic control in loblolly pine [3, 6]. More families should be used in future studies to generalize the findings from QTL analysis to larger populations. A good strategy to make QTL results more generalized is through combining classical genetic mating designs and QTL mapping methods to analyze multiple mapping families [53]. Through a mating design with multiple families, this approach can explore the genetic architecture, as well as the underlying QTL variation in quantitative traits. Verhaegen et al. [53] made use of classic diallel analysis for QTL mapping and found that the power of QTL detection was greatest for the mating design with the fewest but largest families. Hence, using fewer families with larger numbers of individuals per family will raise the efficiency to detect QTL and increase the general utility of QTL results from experimental populations to breeding populations.

As the cost of marker genotyping is going down, the number of families that can be used for QTL mapping is no longer a major constraint for MAS or genomic selection approaches. The power to detect true association between marker and trait will be greatly increased as efficient high-throughput genotyping assays and powerful statistical analysis tools become accessible. As loblolly pine breeding programs enter into new breeding cycles and reach a relatively high level of sophistication, MAS should be considered as one of the important strategies to achieve more genetic gains in shorter time frames.

## Additional file

Additional file 1: Genotypes and phenotypes of all progeny, the definitions of the phenotypes, and the linkage map positions of all mapped SNP loci. (XLSX 352 kb)

## Abbreviations

ADEPT2: Allele discovery of economic pine traits project
CTGN CAP: Conifer translational genomics network coordinated agricultural project
DBH: Diameter at breast height
LOD: Logarithm of odds
LRT: Likelihood ratio test
MAS: Marker-assisted selection
ML: Maximum likelihood interval mapping
MRF: Maximum recombination fraction
QTL: Quantitative trait loci
SNP: Single-nucleotide polymorphism
Strt: Stem straightness
